# The influence of grit on nurse job satisfaction: Mediating effects of perceived stress and moderating effects of optimism

**DOI:** 10.3389/fpsyg.2022.1094031

**Published:** 2023-01-16

**Authors:** Cui Yang, Lu Yang, Dongmei Wu

**Affiliations:** ^1^School of Nursing, Chengdu Medical College, Chengdu, China; ^2^School of Psychology, Chengdu Medical College, Chengdu, China; ^3^Department of Nursing, The Clinical Hospital of Chengdu Brain Science Institute, MOE Key Laboratory for Neuroinformation, University of Electronic Science and Technology of China, Chengdu, China

**Keywords:** nurse, grit, perceived stress, job satisfaction, optimism

## Abstract

**Introduction:**

Nurse job satisfaction, defined as the positive emotional state experienced by nurses regarding their profession, factors related to job performance and outcomes, can affect their career planning and development. Grit, defined as an individual’s relentless effort and enduring enthusiasm for long-term goals, is essential for developing nurses’ competence, increasing job satisfaction, and reducing the willingness to leave the profession. The present study aims to explore the correlation between grit and job satisfaction, whether perceived stress could act as a mediator of the relationship and whether optimism moderated the mediating effect among nurses working in hospitals in southwest China.

**Methods:**

The cross-sectional study utilized self-reported data gathered from 709 nurses in southwest China. To analyze mediating and moderating effects, bootstrapping regressions were conducted.

**Results:**

Perceived pressure mediated the relationship between grit and job satisfaction (indirect effect = 0.195, 95%CI [0.145,0.250]). Furthermore, moderated mediated analysis revealed that optimism moderated grit’s impact on perceived stress (moderating effect = 0.036, 95% CI [0.010, 0.061]).

**Discussion:**

Low levels of grit might reduce nurses’ job satisfaction as their perceived stress levels increase. However, optimism among nurses could diminish this negative effect. Nursing managers should actively seek to improve the grit and optimism of hospital nurses while reducing their perceived pressure, thereby improving job satisfaction.

## Introduction

The shortage and mobility of global nurses are significant challenges (Janiszewski Goodin, 2003). “State of the World’s Nursing 2020” reported a global nursing shortage of approximately 5.9 million in 2018, with a demand gap of 5.7 million by 2030 (Malone, 2021). The World Health Organization estimated that the global nursing shortage will exceed 7.6 million by 2030 (Boniol et al., 2022). This will probably further aggravate the imbalance between supply and demand in the nursing industry, disrupting the medical care system (Haddad et al., 2022). Prior studies have shown that improving nursing job satisfaction can reduce turnover and reverse staffing shortages (Alsubaie and Isouard, 2019; Lee J., 2022). The concept of job satisfaction was first put forward by Hoppock (1935). He argued that job satisfaction refers to the employee’s subjective feelings about their work environment’s psychological and physiological aspects, that is, the degree to which individuals like their jobs. Job satisfaction can impact career planning and career development. Nurses with low job satisfaction are more motivated to leave the field (Gebregziabher et al., 2020; Hu et al., 2022). Moreover, job satisfaction can directly and indirectly affect nursing quality (Kiliç et al., 2022; Wang et al., 2022) and nursing performance (Cho and Kim, 2022). High-quality care is usually associated with high levels of job satisfaction (Dilmaghani et al., 2022). Nurses satisfied with their jobs can effectively perform their duties, improve productivity, and provide high-quality nursing. Low job satisfaction can predict missed nursing care (Al et al., 2022), i.e., the omission or delay of certain activities in patient care (Plevová et al., 2021), which could lead to prolonged hospital stays, ineffective pain management, malnutrition, adverse events (e.g., pressure sores, falls, and hospital-acquired infections), and high mortality (Tschannen et al., 2010; Kalisch and Xie, 2014). Therefore, to reduce nurses’ willingness to leave and provide better patient care, there is an urgent need for hospital managers to improve nurses’ job satisfaction (Makowicz et al., 2022).

### Grit and job satisfaction

With the continuous development of positive psychology and an improved theoretical basis for this field, the focus of research has gradually shifted from identifying defects to exploring the influence of excellent personal traits on individual development. Grit, a positive psychological trait, is of particular interest. Duckworth et al. (2007) initially put forward the concept of grit, defining it as an individual’s unremitting efforts and persistent enthusiasm for long-term goals. On the one hand, grit refers to the determination of an individual to keep working hard even in the face of setbacks. On the other hand, Grit may also represent the tendency of an individual to have a positive interest in constant goals. Grit has proven vital in healthcare, education, and the workplace (Eskreis-Winkler et al., 2014) and is a significant predictor of long-term achievement and success (Aguirre-Urreta and Hu, 2019). Many studies have shown that grit plays an essential role in nurses’ academic performance, mental health, work ability, job satisfaction, and overall performance, which may also reduce staff turnover rate (Jeong et al., 2019; Sellers, 2019; Halperin and Eldar, 2021; Oducado, 2021; Cho and Kim, 2022; Yang and Kim, 2022).

Nurse job satisfaction is a positive emotional state about their occupation, factors related to job performance and job performance results (Lee et al., 2018). Although public attention has focused primarily on the relationship between grit and achievement, grit has also been highlighted as a predictor of nurses’ subjective well-being (Seguin, 2019). Positive personality traits are motivation to promote job satisfaction (Sinniah et al., 2022), and grit as a personality strength affects people’s subjective well-being and is a significant predictor of job satisfaction in particular (Goodman et al., 2017). A meta-analysis (Hou et al., 2022) has shown that grit was positively associated with job satisfaction, suggesting that more extraordinary grit was associated with greater job satisfaction. A study (Dugan et al., 2019) of US salespeople also has shown a significant relationship between grit and job satisfaction, with gritty salespeople performing better and having higher job satisfaction than their less gritty counterparts. Therefore, we focused on the grit of nurses in hospitals in southwest China and hypothesized that their grit was positively related to their job satisfaction in this study.

### Perceived stress, grit, and job satisfaction

Perceived stress refers to an individual’s subjective feeling or cognitive evaluation of stressful events (Cohen et al., 1983). Robbins’ stress model (Robbins, 2003) divides stressors into environmental, organizational, and personal factors. Personal factors include family problems, financial problems, and personality traits such as grit. Stress manifests itself in various ways in each individual, where the psychological effects include anxiety, depression, and reduced job satisfaction. According to Robbins, personality traits may be responsible for an individual’s increased stress, which, in turn, can cause a decrease in job satisfaction. Therefore, we hypothesized that perceived stress might play a mediating role in the relationship between grit and job satisfaction.

Nurses faced greater psychological distress and pressure during COVID-19 (Xia et al., 2021). The theory on the relationship between grit and health (Kobasa and Puccetti, 1983) holds that grit is a protective factor for people dealing with stressful events. Grit is negatively related to negative emotions (Clement et al., 2020; Lee M., 2022), and individuals who persevere tend to have more positive emotions (Lee M., 2022), stronger psychological resilience (Tang et al., 2022), are more likely to receive social support, and have a higher level of hope (Yang and Wu, 2021). Nurses with low grit have been found to have higher stress levels and more mental health problems, such as anxiety, depression, and other harmful emotions (Peng and Wu, 2022).

Moreover, prior literature (Piotrowski et al., 2022) has reported that perceived stress has a significant negative impact on nursing job satisfaction, and the higher the perceived stress, the lower the job satisfaction. Failure to deal with stress appropriately can lead to various mental health problems (Yang et al., 2022). Nurses often face nurse–patient disputes, medical disputes, and patient attacks and have difficulty balancing work and family. These nurses receive less positive feedback from their work, and their anxiety is prominent. In addition, job burnout is a response to long-term emotions and interpersonal workplace stressors (Maslach et al., 2011), and greater levels of perceived pressure are more likely to produce job burnout. Job burnout is also the most significant predictor of low job satisfaction (Shanafelt et al., 2009). Therefore, we further predicted that perceived stress is a mediator of the relationship between grit and job satisfaction among hospital nurses in southwest China.

### Optimism, grit, and perceived stress

Not all people with low grit report an increase in perceived stress. Many helpful factors may help prevent low grit from turning into stress. One example is the important psychological resource of optimism, prolonged, across-scenario, and steady dispositional tendency to anticipate good future outcomes (Scheier et al., 1986). The scientific definition of optimism focuses on expectations about the future. Expectancy-value theories reflect the importance of confidence in achieving goals (Wigfield and Eccles, 2000). Those who believe they will eventually achieve results and have faith in their goals will continue to persevere, even in the face of great adversity. If people doubt they can reach their goals, they may give up on their efforts. They may stop prematurely, or they may not actually start moving. Optimism and pessimism, universal versions of self-confidence and doubt, are about living with confidence and doubt, not simply a particular context. Optimists hence tend to be self-confident and persistent in the face of various life challenges (Carver et al., 2010), which explains why optimism promotes and predicts perseverance, an essential component of grit (Binsch et al., 2017).

According to the conservation of resources theory (COR), individuals can mobilize resources to cope with resource loss (Hobfoll, 1989). A study discovered that individuals with high levels of optimism were prone to higher grit levels (Agrawal et al., 2022). Optimism tends to evaluate trends and outcomes of things positively, providing a positive outlook on the efforts people make in the face of difficulties (Scheier and Carver, 1985). Many studies have found a link between higher levels of optimism and lower levels of stress (Öcal et al., 2022; Puig-Perez et al., 2022). Optimism can act as a dynamic process to moderate the harmful effects of adversity (Brydon et al., 2009). Given the above theories and studies, this study assumed that optimism would moderate the association between grit and perceived stress.

How to enable nurses to have high levels of job satisfaction is a significant issue with lasting and profound implications. Thus, exploring the impact of personal variables that modulate or interfere with nurses’ job satisfaction is necessary. We aimed to investigate the relationship between grit, perceived stress, optimism, and job satisfaction of hospital nurses in southwest China and to examine the mediating role of perceived stress between nurses’ grit and job satisfaction, as well as the moderating role of optimism. Our study tried to hypothesize the following:

*Hypothesis 1*: Nurses’ grit would be positively correlated to their job satisfaction.

*Hypothesis 2*: Nurses’ perceived stress would mediate the relationship between grit and their job satisfaction.

*Hypothesis 3*: Nurses’ optimism would moderate the relationship between grit and their perceived stress.

### Hypothetical research model

**Figure 1:**
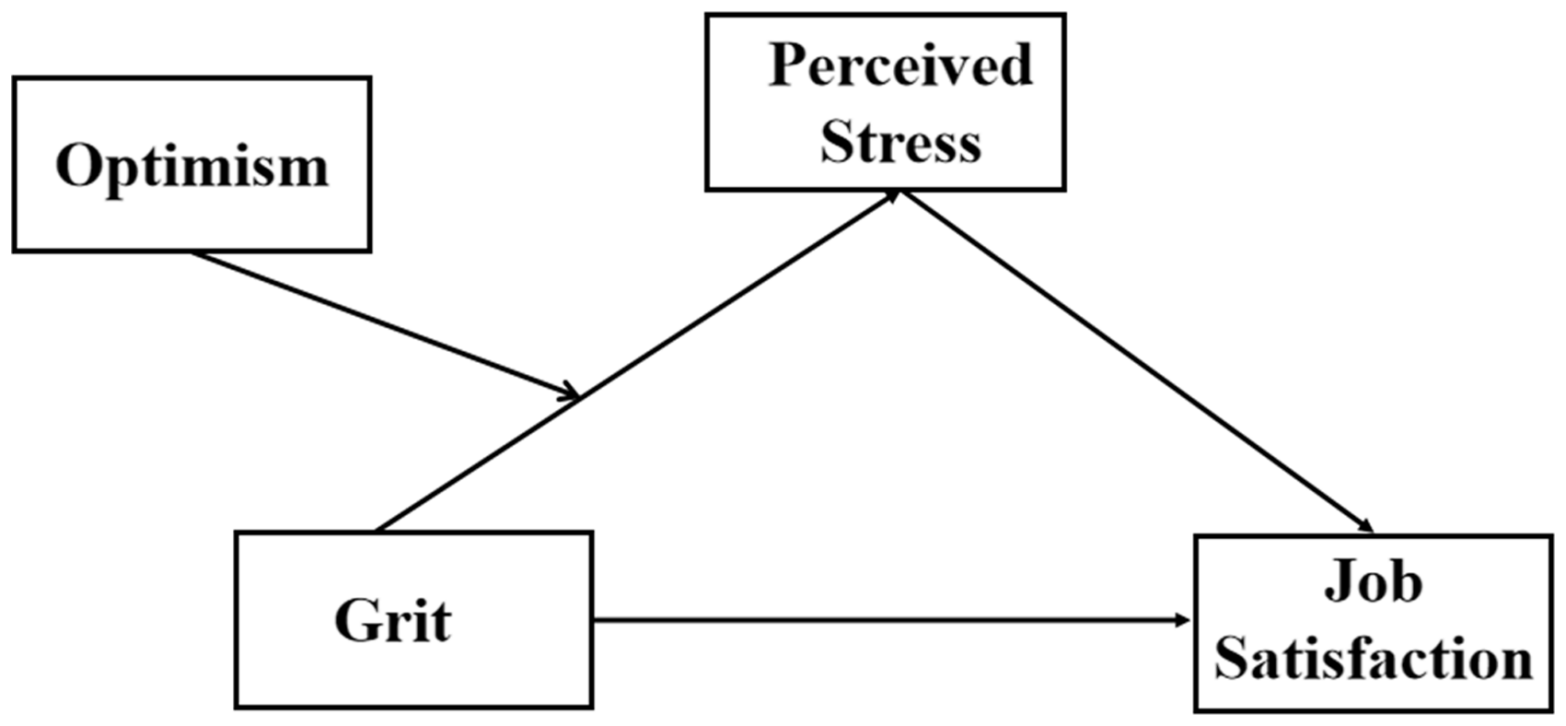
The hypothesized moderated mediation model.

In this study, we focused on the impact of grit on job satisfaction among nurses in Southwest China. Moreover, we explored the potential mechanism of this connection with perceived stress as a mediator and optimism as a moderator. Our study formulated the below hypotheses (see Figure 1).

## Materials and methods

### Participants and procedure

**Figure 2:**
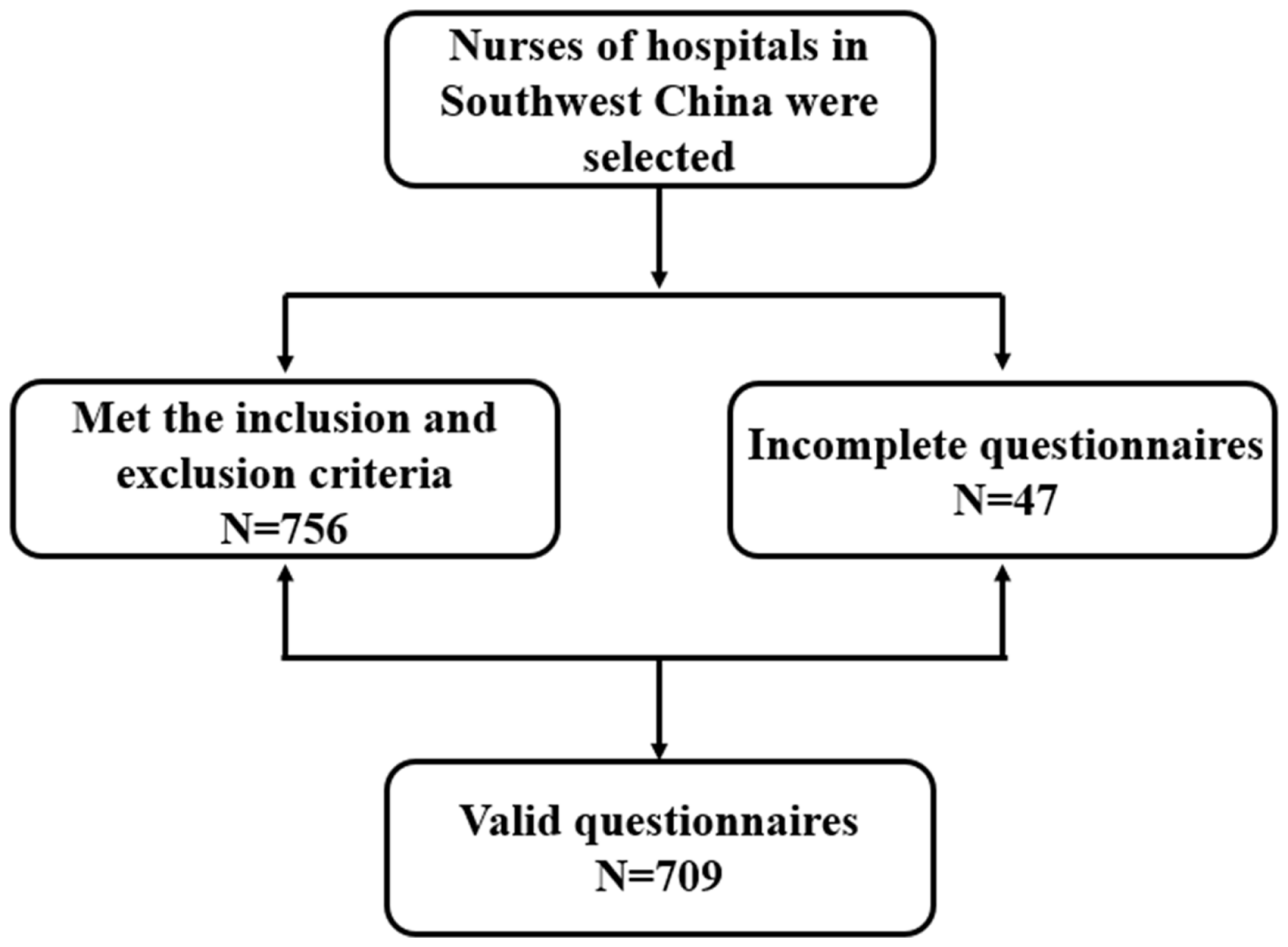
The step flow diagram including all steps from screening to study.

The cross-sectional study used an anonymous internet-based questionnaire to select nurses of hospitals in southwest China by convenience sampling methods from January 2021 to May 2022. Nurses who submitted the online survey were considered to have agreed to participate in this study. The Inclusion criteria were: (a) obtained the nurse qualification certificate from China; (b) at least 1 year of experience working in clinical nursing or clinical nursing management; (c) no previous diagnosis of psychosis or drug or alcohol dependence; (d) have basic telephone or computer skills; and (e) volunteered to participate in this study and sign the informed consent form. Exclusion criteria were: (a) Those who go out or come to the hospital for further study and (b) Those who practice or train nurses. After obtaining the consent of the relevant person in charge of the hospital, an investigator from each unit was selected to perform unified training. To ensure sufficient sample size, we performed a power analysis of the multiple regression with G*power, using a medium effect size (*f*^2^ = 0.15), an alpha value of 0.05, and a power of 0.95 (Faul et al., 2009). The estimated sample size was 190–207 after considering the 10–20% of incomplete surveys. A total of 756 nurses met the inclusion and exclusion criteria and 709 valid responses were collected, giving a valid return rate of 93.7%. Figure 2 shows all steps from screening to study. The Ethics Committee of the Fourth People’s Hospital of Chengdu granted this study, and its clinical trial registration number in China was ChiCTR1900020715.

### Measures

#### Sociodemographic characteristics data

The general data questionnaire was designed by the researchers and included age, length of nursing work, gender, marital status, educational background, and nurse title. Age (in years), Length of nursing work (in years), gender (0 = female, 1 = male), marital status (0 = single, 1 = married, 2 = divorced, and 3 = widowed), educational background (1 = Technical secondary school, 2 = college degree, 3 = bachelor degree, and 4 = graduate degree), and nurse title (1 = None, 2 = Primary, 3 = Intermediate, and 4 = Vice senior) were all control variables. In China, nurses are divided into non-titled nurses (similar to nursing assistants), primary nurses (similar to licensed practical nurses), intermediate nurses (similar to registered nurses), and vice senior and senior (similar to advanced practice registered nurses).

#### Grit

The Short Grit Scale (Grit-S), developed by Duckworth and Quinn (2009), consists of two dimensions (perseverance of effort and consistency of interest) with four items each. It is graded from 1 (completely inconsistent) to 5 (completely consistent) on a five-grade scale (the items of consistency of interest dimension are reversed). The average of the eight answers represents the respondent’s courage. The higher the total score, the higher the level of grit. The Chinese version of the Grit-S has good reliability and validity and is widely used in China (Canario et al., 2018; Zhong et al., 2018; Luo et al., 2020). The scale has been verified in Chinese nurses, with Cronbach α coefficients for the total scale and the two dimensions of 0.68–0.75 (He et al., 2021).

#### Perceived stress

The Perceived Stress Scale (PSS-10) was compiled by Cohen et al. (1983). In 1988, four items from the scale were deleted to form a 10-item short scale (PSS-10), which is currently one of the most commonly used research tools for measuring perceived stress. The scale is scored using Likert’s five points (0 = never, 1 = almost never, 2 = sometimes, 3 = often, and 4 = always). The PSS-10 consists of six negative projects and four positive Questions. It is scored from 0 to 40, with higher scores representing increased perceived stress. The PSS-10 has good psychometric characteristics in the Chinese population (Wu et al., 2018). The Cronbach’α coefficients of the total scale and its two dimensions were 0.816–0.901, with good reliability and validity. The PSS-10 can also be used to detect nurse psychological tension (Min-rou et al., 2022).

#### Optimism

The Life Orientation Test-Revised (LOT-R) was compiled by Scheier and Carver (1985) and revised in 1994 (Scheier et al., 1994). This scale is used to measure participants’ optimism (Zhang et al., 2020). It has 10 items, including three items of optimism, three items of pessimism, and four non-scoring cover-up filling questions. Masking questions are not scored, and other items are scored by the Likert five-grade method, with 0 representing “very different opinions” and 4 representing “in complete agreement.” The total score ranges from 0 to 24. The higher the score, the higher the patient’s optimism level. The LOT-R showed good reliability and validity in the Chinese cultural context, and the Cronbach’α coefficients was 0.739 (Tang et al., 2020). Cronbach’α for the present sample was 0.670.

#### Job satisfaction

The Job Satisfaction Scale (JS), put forward by Schriesheim and Tsui (1980), uses six items to form an index to describe comprehensive job satisfaction. It also examines overall job satisfaction in six respects: job intensity and pressure, leadership relationship, colleague relationship, remuneration, promotion opportunities, and overall satisfaction. Example questions include: “your satisfaction with your salary.” The JS is scored using five-point scoring, ranging from “very dissatisfied” to “very satisfied.” The higher the score, the higher the job satisfaction. The Cronbach’α coefficient of the JS was 0.857, which is suitable for testing nurses.

### Statistical analysis

This study utilized IBM SPSS 26.0 (Armonk, NY, United States) for data analysis. Continuous data were represented as ^−^*x* ± *s* and categorical as (*n*) and percentage (%). Pearson’s correlation analysis was used to identify relationships between variables. Based on Hayes’ PROCESS macro program, we used bootstrapping to test the significance of the mediated models. To test the indirect effects of grit on job satisfaction through perceived stress, we used Model 4. To test the moderated mediation, we used Hayes’ PROCESS macro Model 7. Bootstrap confidence intervals (CIs) ascertain whether the effects of Model 4 and Model 7 are statistically significant on the basis of 5,000 random samples. The effect was considered significant if the CIs did not contain zero.

## Results

### Common method biases test

We used Harman’s single-factor test for all questions on the four scales before data processing. We discovered that the first common factor analyzed explained only 25.20% (<40%) of the variance, indicating that although the questionnaire was used in this study, the common method bias is not apparent.

### Descriptive data and Pearson’s correlations

The sociodemographic characteristics are shown in Table 1. A total of 709 nurses qualified for this study, including 643 females (90.7%) and 66 males (9.3%). Their average age was 31.74 ± 7.38 years. They worked a mean 10.67 ± 8.12 years. Their marital status was 198 unmarried (27.9%), 483 married (68.1%), 26 divorced (3.7%), and 2 widowed (0.3%). Thirty-seven nurses attended technical secondary schools (5.2%), 668 university (94.2%), and 4 graduate school (0.6%). With respect to nurse title, 163 (23.0%) had no job title, 366 (51.6%) were primary nurse title, 170 (24.0%) were intermediate nurse title, and 10 (1.4%) were vice senior.

**Table 1:** Sociodemographic information of the participants (*n* = 709).

Variable	Mean ± SD (range) *N* (%)
Age	31.74 ± 7.38 (18–55)
Length of nursing work	10.67 ± 8.12 (0–39)
Gender	
Male	66 (9.3%)
Female	643 (90.7%)
Marital status	
Unmarried	198 (27.9%)
Married	483 (68.1%)
Divorced	26 (3.7%)
Widowed	2 (0.3%)
Education background	
Technical secondary school degree	37 (5.2%)
College degree	270 (38.1%)
Bachelor degree	398 (56.1%)
Graduate degree	4 (0.6%)
Nurse title	
None	163 (23.0%)
Primary	366 (51.6%)
Intermediate	170 (24.0%)
Vice senior	10 (1.4%)

SD, Standard Deviation.

Table 2 shows the results of the Pearson’s correlations. The results indicated that grit (*r* = 0.293, *p* < 0.01), perceived stress (*r* = −0.430, *p* < 0.01), and optimism (*r* = 0.234, *p* < 0.01) were associated with job satisfaction. Grit was negatively related to perceived stress (*r* = −0.502, *p* < 0.01) and positively correlated with optimism (*r* = 0.378, *p* < 0.01). Optimism was negatively associated with perceived stress (*r* = −0.442, *p* < 0.01). Therefore, Hypothesis 1 was validated.

**Table 2:** Bivariate correlation.

	M ± SD	1	2	3	4
1. Grit	27.03 ± 4.141	1			
2. Perceived stress	26.99 ± 5.583	−0.502^**^	1		
3. Optimism	21.94 ± 2.962	0.378^**^	−0.442^**^	1	
4. Job satisfaction	21.07 ± 4.244	0.293^**^	−0.430^**^	0.234^**^	1

M, Mean. SD, Standard Deviation. ^**^p < 0.01.

### Mediating effect analysis

Table 3 presents the indirect impact of grit on job satisfaction *via* perceived stress. After we controlled for the effects of age, gender, etc., the indirect mediated effect of grit on job satisfaction was significant (β = 0.195, CI [0.145, 0.250]). Grit was a significantly positive predictor of job satisfaction (β = 0.299, CI [0.226, 0.371]). Grit was a significantly negative predictor of perceived stress (β = −0.672, CI [−0.759, −0.586]), while perceived stress had a significantly negatively predicted effect on job satisfaction (β = −0.290, CI [−0.348, −0.231]). Moreover, the direct effect of grit on job satisfaction was still significant (β = 0.104, CI [0.025, 0.183]) when mediating variables were added. Additionally, the upper and lower bounds of the bootstrap 95% CI for the direct effect of grit on job satisfaction and the mediating effect of perceived stress did not include 0, indicating that the mediating effect was significant. The mediation effect accounted for 65.22% of the total effect, which confirmed that perceived stress partially mediated the relationship between grit and job satisfaction. Therefore, Hypothesis 2 was proved.

**Table 3:** Mediation effect of perceived stress on the relationship between grit and job satisfaction (*n* = 709).

Outcome	Mediation analysis paths	Estimated	95% bias-corrected CI		Proportion
			LLCI	ULCI	
Job satisfaction	Total effect	0.299^***^	0.226	0.372	
	Direct effect	0.104^**^	0.025	0.183	34.78%
	Indirect effect	0.195^***^	0.145	0.250	65.22%
	Grit → Perceived Stress	−0.672^***^	−0.759	−0.586	
	Perceived Stress → Job satisfaction	−0.290^***^	−0.348	−0.231	

Age, length of nursing work, gender, marital status, educational background, and nurse title were used as control variables. CI, Confidence Interval. ^**^p < 0.01. ^***^p < 0.001.

### Moderated mediation effect analysis

**Figure 3:**
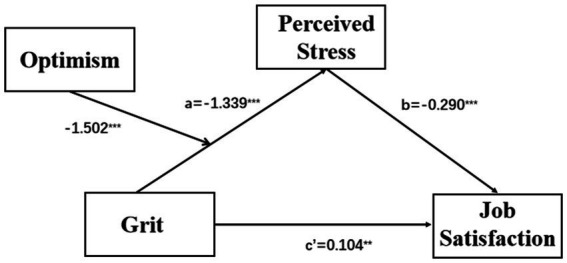
The final moderated mediation model (^∗∗^*p* < 0.01; ^∗∗∗^*p* < 0.001).

**Figure 4:**
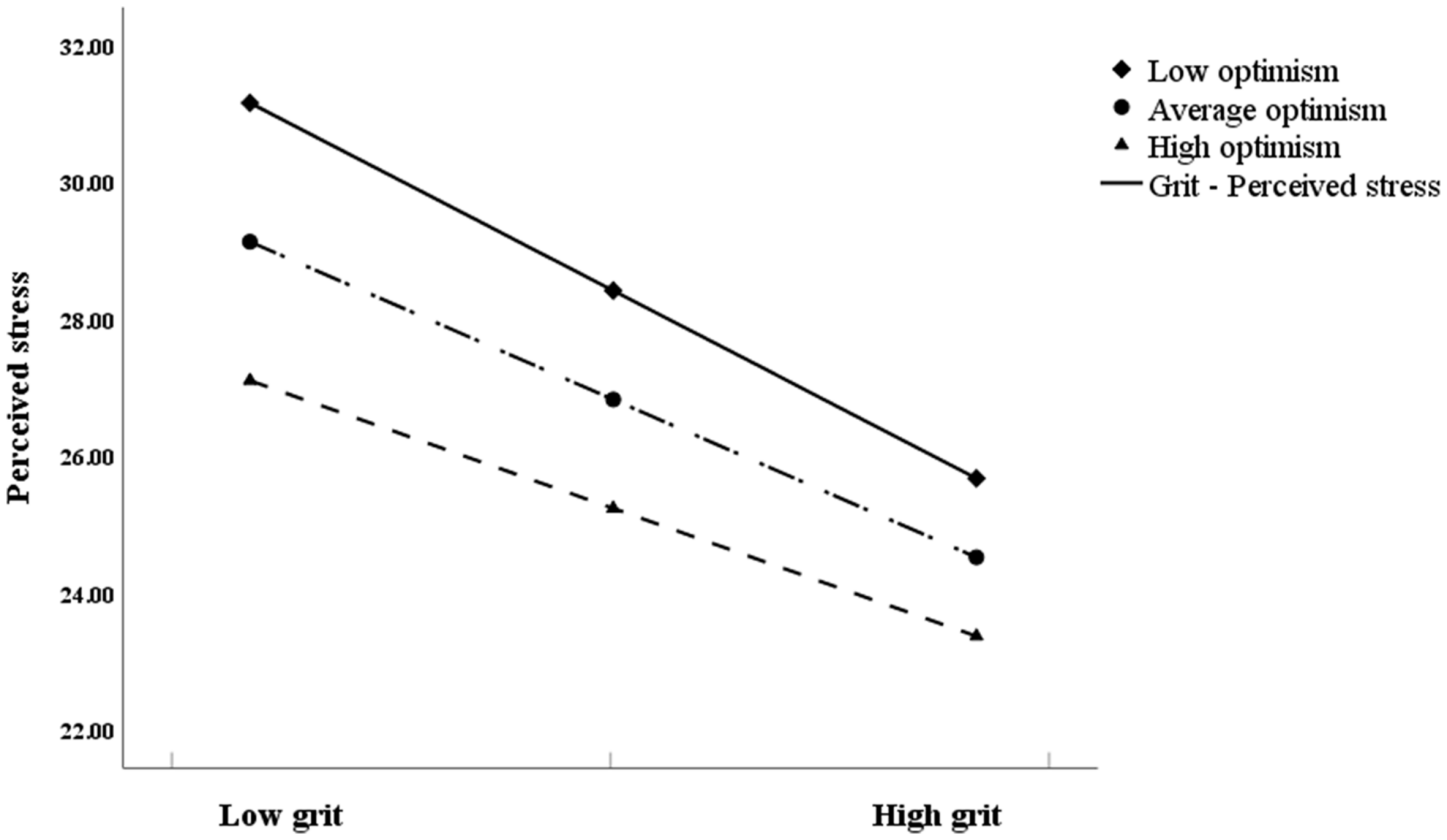
The moderation plot for optimism.

Hypothesis 3 forecasted optimism would moderate the first half of the indirect effect of Hypothesis 2. Figure 3 shows the final moderated mediation model. The indexes of moderated mediation were significant (see Table 4; β = 0.004, 95% CI [−0.019, −0.002]), suggesting that the effect of grit on perceived stress varied with optimism. In the moderated mediation model, the interaction term grit × optimism was significant (see Table 4; β = 0.036, 95% CI [0.010, 0.061]). To interpret the interaction results, we plotted the interaction effects in Figure 4. Grit had a greater impact on perceived stress for nurses with poor optimism than those with high optimism. In addition, we presented the influence of the moderator at different values (1 SD below the mean, the mean, and 1 SD above the mean). Nurses with low, average, and high levels of optimism all showed significant effects (see Table 5; effect_lowlevel_ = −0.662, *t* = −9.973, and *p* < 0.001; effect_averagelevel_ = −0.557, *t* = −12.038, and *p* < 0.001; effect_highlevel_ = −0.451, *t* = −8.518, and *p* < 0.001). Therefore, Hypothesis 3 was confirmed.

**Table 4:** Results of the moderated mediation model.

Variable	Mediator	Dependent variable
	Perceived stress	Job satisfaction
Index of moderated mediation	−0.010 [−0.019, −0.002]	-
Grit	−1.339 [−1.924, −0.755]^***^	0.104 [0.025, 0.183]^**^
Perceived stress	-	−0.290 [−0.348, −0.231]^***^
Optimistic	−1.502 [−2.198, −0.806]^***^	-
Girt × Optimistic	0.036 [0.010, 0.061]^**^	-
R-square	0.341	0.203

Age, length of nursing work, gender, marital status, educational background, and nurse title were used as control variables. ^**^p < 0.01; ^***^p < 0.001.

**Table 5:** Mediating effect of perceived stress conditional on optimism.

Moderator value	Mediating effect	*t*	LLCI	ULCI
Low optimism	−0.662	−9.973^***^	−0.793	−0.532
Average optimism	−0.557	−12.038^***^	−0.647	−0.466
High optimism	−0.451	−8.518^***^	−0.555	−0.347

Age, length of nursing work, gender, marital status, educational background, and nurse title were used as control variables. CI, confidence interval. ^***^p < 0.001.

## Discussion

The present study used a moderated mediation to examine whether perceived stress would mediate the link between nurses’ grit and job satisfaction and whether optimism support would moderate the relationship between nurses’ grit and perceived stress. The findings revealed that nurses’ grit was positively associated with their job satisfaction (see Hypothesis 1), while perceived stress could mediate this association (see Hypothesis 2). Moreover, optimism could moderate the association between grit and perceived stress among nurses in Southwest China (see Hypothesis 3).

### Grit and job satisfaction

First, according to the results of this study, we found grit has an essential impact on job satisfaction among nurses in Southwest China, which agrees with previous studies showing that high grit could result in high job satisfaction (Ko and Kim, 2019; Cho and Kim, 2022). As a result, while constantly striving to achieve their goals without giving up, individuals positively influence and increase their satisfaction. However, this study differed from the literature study (Reed et al., 2012) of family physicians in Idaho, United States, which found no association between grit and job satisfaction, possibly because the specificity of the survey population and survey area hindered this relationship. Idaho is a rural state largely isolated from the urban environment. It demands a high level of grit from all physicians, so physicians with high and low job satisfaction reported the same grit on the grit scale.

### The mediating role of perceived stress

As hypothesized, our study suggested that perceived stress could mediate the relationship between grit and job satisfaction, which may reveal the potential mechanism regarding how grit indirectly influences job satisfaction. According to the self-determination theory (Franke et al., 2000), people as active creatures have the potential for self-realization and self-growth. Individuals can promote healthy growth and self-development by satisfying their basic psychological needs. Existing research shows that perseverant individuals may develop coping mechanisms for promoting happiness and hope when faced with difficulties (Musso et al., 2019), and driven by such positive emotions, individuals may feel less stressed, may recover more easily from negative events, and ultimately maintain a higher level of job satisfaction. According to the person-environment fit theory (Caplan, 1987), a mismatch between the perceived environment and personal characteristics, such as adversity, may lead to negative emotions and stress in people with low levels of grit. Furthermore, excessive job stress may increase turnover and reduce job satisfaction (Lim et al., 2022). Practicing medical care during a pandemic is a challenge for nurses (Tyer-Viola, 2019) and can result in higher occupational stress and low job satisfaction (Kwiecień-Jaguś et al., 2018; Leskovic et al., 2020). Conversely, medical professionals with a higher degree of fit have higher job satisfaction (Sinniah et al., 2022).

### The moderating role of optimism

Our study also investigated whether optimism moderated the correlation between grit and perceived stress among nurses in hospitals in Southwest China. We found optimism to play a moderating role in the first half of the mediated pathway “grit → perceived stress → job satisfaction,” suggesting that optimism could influence nurses’ job satisfaction by moderating the relationship between levels of grit and perceived stress and hence job satisfaction. Optimism was found to be a protector of perceived stress among nurses, supporting the previous findings (Puig-Perez et al., 2022). According to the stress buffer hypothesis, positive individual factors can act as a cushion against stress and enable individuals to cope better. As a positive psychological factor or personality construct, optimism tends to maintain a consistently positive expectation of future consequences, even in uncertainty and adversity (Scheier et al., 1994). Optimism is associated with many positive outcomes, such as better mental health and job satisfaction (Zhang et al., 2021). Nurses’ optimism is positively linked with approach coping strategies to eliminate, diminish or manage stressors or emotions (Zhang et al., 2020). The coping style of optimists, i.e., their unique method orientation to managing stressful problems and stress-induced emotions, would help them achieve a sense of well-being (Nes and Segerstrom, 2006), which explains the ability of optimists to adapt to stressful or negative events. Previous research has shown that grit is enhanced through the positive psychological factor of optimism (Tenney et al., 2015), and the impact of negative emotions on stress is moderated (Rahman et al., 2022). Therefore, nursing managers and educators need to adopt effective interventions to foster optimistic psychological traits in nurses and nursing students to alleviate burnout and workplace stress among nurses.

### Implications

This study enriches positive psychology and may provide a reference for future programs to improve nurses’ job satisfaction. Moreover, the evidence obtained in the study may contribute to developing different strategies and policies related to the construction of quality care and safe care programs and reducing adverse health outcomes of clinical patients.

### Priorities and next steps

Based on these findings, we make the following recommendations. Firstly, nurses need to be helped to develop the quality of grit, enhance job satisfaction, reduce turnover rates, and ultimately redress the imbalance between supply and demand in the nursing profession. Secondly, hospital managers should provide nurses with adequate support when they are at high levels of perceived stress, listen carefully to their demands, and teach them appropriate ways of releasing stress. Thirdly, nursing managers should foster optimism among nurses, thereby alleviating perceived stress. Fostering grit in nurse managers and clinical nurses is critical to maintaining their interest in emergency preparedness and ensuring that the needs of patients and caregivers are met when disasters such as COVID-19 occur (Tyer-Viola, 2019). We recommend that nursing managers pay particular attention to grit and optimism when selecting nursing talent. Educators in medical schools should also strengthen the focus on grit and optimism in nursing training.

### Limitations

This present study has several limitations. Firstly, as this is a cross-sectional survey, it cannot elucidate the causal relationships between these variables. Further longitudinal studies are needed to verify the validity and reliability of our findings. Secondly, this study’s mediating and moderating variables were perceived stress and optimism, and other variables that were not assessed may also affect nurses’ psychological state and job satisfaction. Finally, the population of this study was nurses in southwest China, affecting the external applicability of our results.

## Conclusion

In summary, our study examined the mediating role of perceived stress and the moderating role of optimism on the relationship between grit and job satisfaction among hospital nurses in southwest China and showed that nurses’ job satisfaction could be influenced by grit through perceived stress, while optimism could act as a moderator on the pathway from grit to perceived stress.

## Data availability statement

The raw data supporting the conclusions of this article will be made available by the authors, without undue reservation.

## Ethics statement

The studies involving human participants were reviewed and approved by the Ethics Committee of the Fourth People’s Hospital of Chengdu, China. The patients/participants provided their written informed consent to participate in this study.

## Author contributions

CY, LY, and DW contributed to this manuscript’s conceiving, researching, and writing. All authors contributed to the article and approved the submitted version.

## Funding

This work was supported by the Sichuan Science and Technology Program (grant no. 2018JY0306) and the National Natural Science Foundation of China (grant no. 82001444).

## Conflict of interest

The authors declare that the research was conducted without any commercial or financial relationships that could be construed as a potential conflict of interest.

## Publisher’s note

All claims expressed in this article are solely those of the authors and do not necessarily represent those of their affiliated organizations, or those of the publisher, the editors and the reviewers. Any product that may be evaluated in this article, or claim that may be made by its manufacturer, is not guaranteed or endorsed by the publisher.
